# Calcaneocuboid and Naviculocuneiform Dislocation: An Unusual Injury of the Midfoot

**DOI:** 10.1155/2020/8818823

**Published:** 2020-09-28

**Authors:** Anne Kummer, Xavier Crevoisier, Antoine Eudier

**Affiliations:** ^1^Hôpital Intercantonal de la Broye (HIB), Payerne, Switzerland; ^2^Lausanne University Hospital (CHUV), Switzerland

## Abstract

*Introduction*. Midfoot dislocations are rare traumatic injuries. The best known patterns involve the Lisfranc and Chopart joints, although some other types have been described. Dislocations that occur at the level of the naviculocuneiform and calcaneocuboid joints simultaneously represent a very rare configuration of dislocation. *Case Presentation*. A 34-year-old man sustained a crush injury to his left foot causing a complete dislocation through the naviculocuneiform and calcaneocuboid joints. Immediate closed reduction and percutaneous pinning were performed, followed by open reduction and stabilization of both joints two weeks later. Anatomical reduction was obtained, and the clinical outcome remained satisfactory 10 months after surgery. *Discussion*. Anatomical reduction is essential to obtain favorable outcomes in traumatic midfoot injuries. An unusual pattern of midfoot dislocation can be treated according to the same principles as those for classical Lisfranc or Chopart injuries.

## 1. Introduction

Traumatic injuries and dislocations of the midfoot are commonly referred to as Chopart or Lisfranc injuries, related to an eponymous joint. The Chopart joint takes its name from François Chopart who practiced amputations at the level of the talonavicular and calcaneocuboid joints in the 18^th^ century [1]. The Lisfranc joint is related to Jacques Lisfranc who described amputations through the tarsometatarsal joints in the 19^th^ century [2]. Chopart injuries are rare [3, 4], and dislocation of this joint is even more so [5]. These injuries most often result from high-energy trauma such as motor vehicle accidents or crush injuries [6], from a heavily loaded distortion of the midfoot in supination-flexion or from an axial load along the medial column of the foot [1]. Fractures and dislocations of the Chopart joint have been classified by Main and Jowett [3] in several types. Dislocations of the naviculocuneiform joint represent also an unusual form of midfoot injury [7] and are most often described in association with or as a variant of injuries to the Lisfranc joint [8, 9], or as isolated dislocation of the medial and/or intermediate cuneiforms [7, 10–12].

We report a case of unusual dislocation of the midfoot, since the dislocation pattern occurred through the calcaneocuboid and naviculocuneiform joints.

## 2. Case Presentation

A 34-year-old man presented to the emergency department after sustaining a traumatic injury to his left foot. He reported that a Caterpillar machine rolled on his left leg from behind, while he was working on a construction site. The physical examination revealed a severe abduction deformity of his left foot together with an important swelling of the foot, ankle, and lower leg. Vascular and neurological examination was normal.

**Figure 1:**
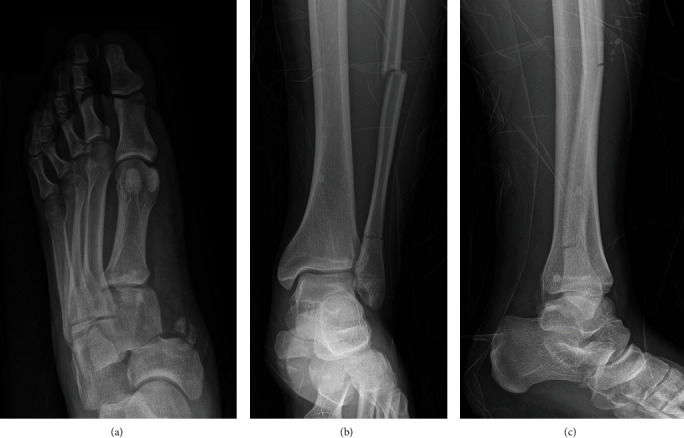
(a) Anteroposterior X-rays of the left foot show a dislocation through the naviculocuneiform joint with bony avulsions. (b) Frontal and (c) lateral X-rays of the ankle show a bifocal fracture of the fibula.

**Figure 2:**
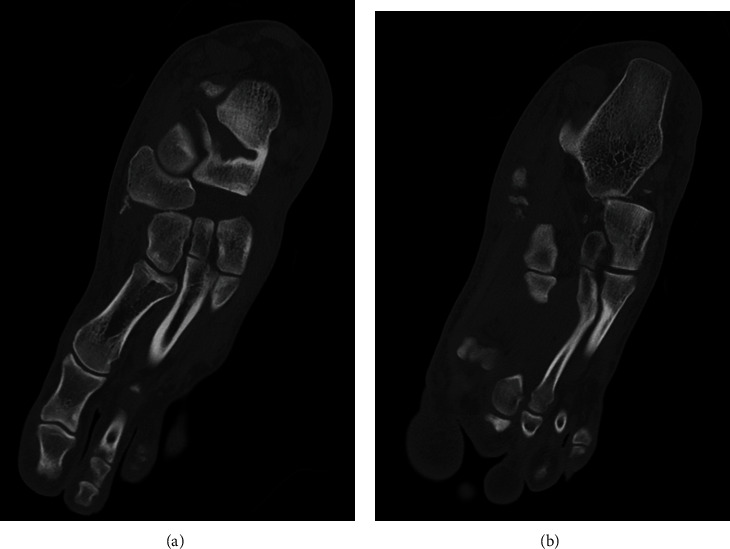
Oblique multiplanar reconstruction CT of the left foot shows dislocation of the (a) naviculocuneiform joint and of the (b) calcaneocuboid joint.

Standard X-rays showed a dislocation between the navicular and the three cuneiforms, a lateral displacement of the cuboid (Figure 1(a)), and a bifocal fracture of the fibula (Figures 1(b) and 1(c)). Computed tomography of the foot confirmed the naviculocuneiform dislocation with an associated calcaneocuboid dislocation, as well as a marginal fracture of the medial cuneiform and an avulsion fracture of the anterior calcaneal process (Figure 2).

**Figure 3:**
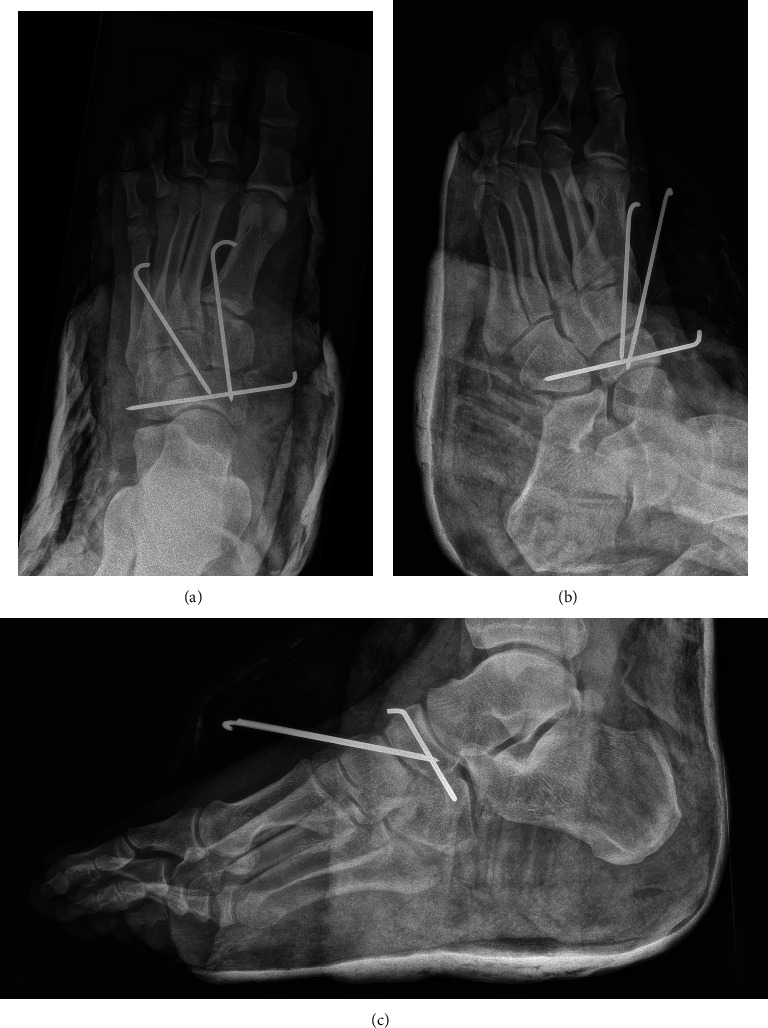
(a) Anteroposterior, (b) oblique, and (c) lateral X-rays of the left foot after closed reduction and percutaneous pinning.

**Figure 4:**
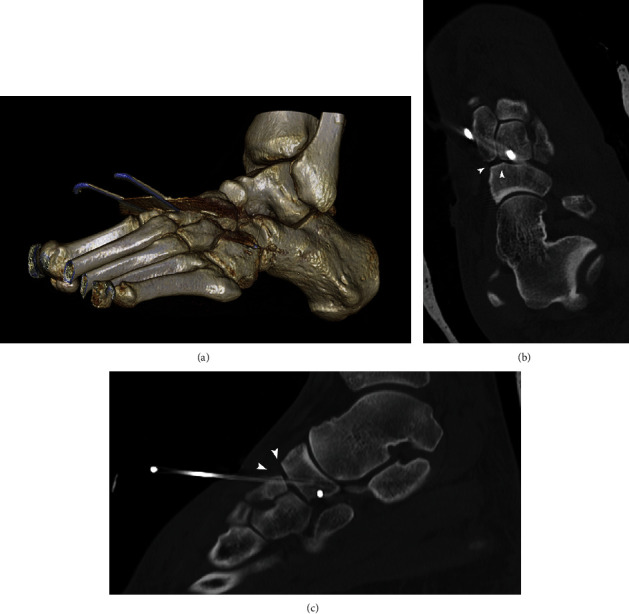
(a) Dorsolateral volume-rendered CT of the left foot shows the incomplete reduction of the calcaneocuboid joint. (b) Oblique and (c) lateral multiplanar reformatted CT show the incomplete naviculocuneiform reduction (white arrowheads).

The patient was taken to the operating room for closed reduction of the naviculocuneiform and calcaneocuboid joints. Due to residual instability, percutaneous pinning was performed between the navicular and the medial cuneiform (N-C1), between the navicular and the intermediate cuneiform (N-C2), and between the cuboid and the navicular (Figure 3). A second computed tomography was performed after the reduction, showing incomplete reduction between the cuboid and the calcaneus (Figure 4(a)) as well as at the level of the naviculocuneiform joint (Figures 4(b) and 4(c)).

**Figure 5:**
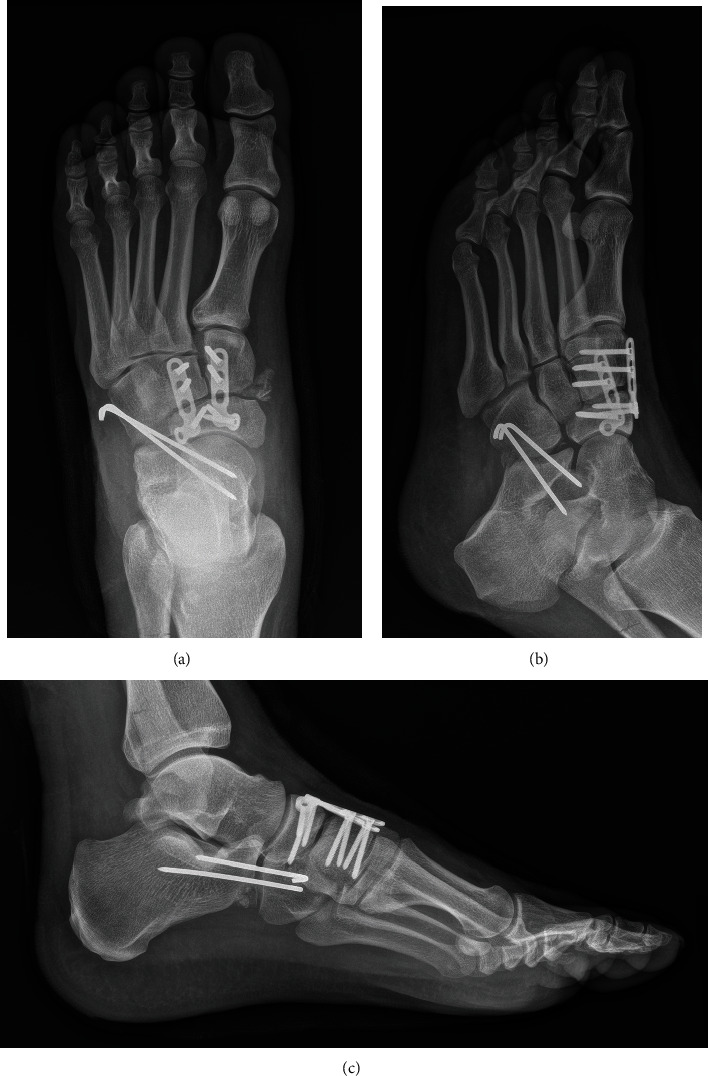
(a) Anteroposterior, (b) oblique, and (c) lateral X-rays of the left foot after open reduction, showing internal fixation of N-C1 and N-C2 joints, and pinning of the calcaneocuboid joint.

Two weeks later, open reduction was performed. A dorsomedial incision was carried out; the N-C1 joint was exposed through the interval between the tibialis anterior and the extensor hallucis longus, and the N-C2 joint through a second interval lateral to the extensor hallucis longus. Optimal reduction was obtained under direct vision, and fixation was performed using two 2.7 locking plates (N-C1 and N-C2). A second incision was performed, extending from the sinus tarsi to the base of the 5^th^ metatarsal. The origin of the extensor digitorum brevis was partially detached to expose the calcaneocuboid joint. After reduction, percutaneous pinning of the calcaneocuboid joint was performed. Postoperative X-rays showed optimal reduction (Figure 5). The bifocal fracture of the fibula was not part of an ankle fracture mechanism but resulted from a direct impact injury. Therefore, and considering its correct alignment, it was treated conservatively.

Postoperative measures included bed rest for 48 hours; then, partial weight-bearing was initiated for 6 weeks with a short leg cast. The calcaneocuboid Kirschner wires were removed at 6 weeks, and progressive weight-bearing was authorized. The patient was advised to wear a rigid sole shoe for one year.

**Figure 6:**
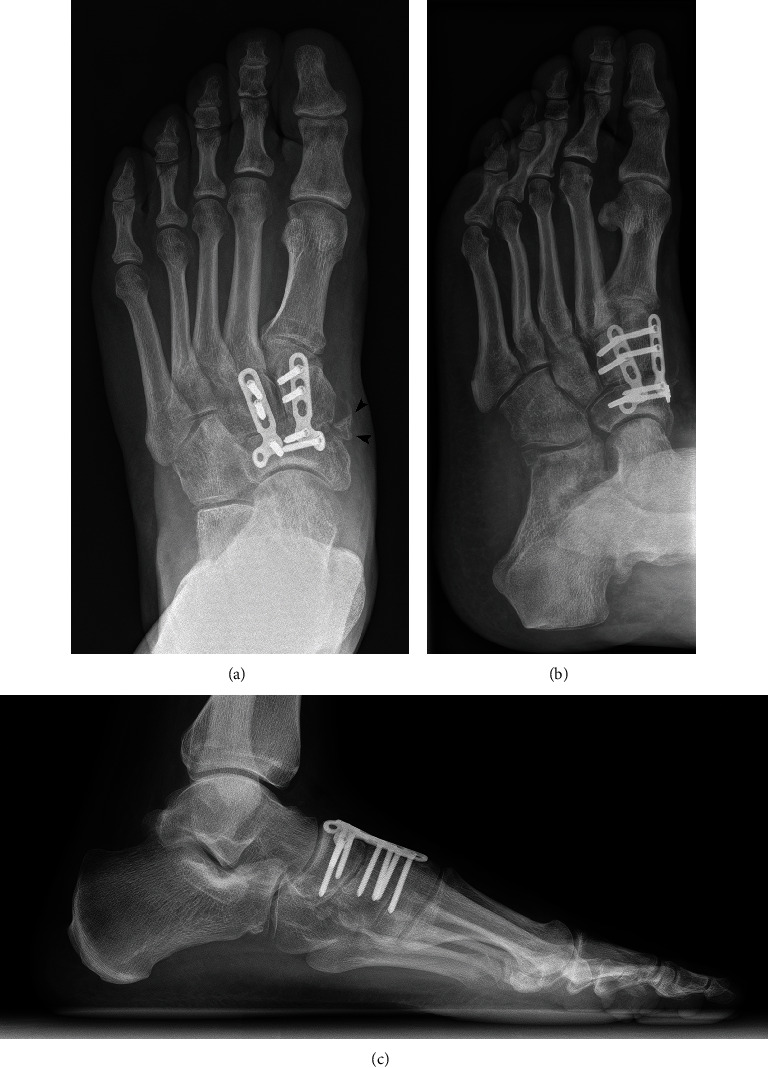
(a) Anteroposterior weight-bearing, (b) oblique, and (c) lateral weight-bearing X-rays of the left foot 10 months after the surgery. Black arrowheads show the osseous fragment next to the N-C1 joint responsible for the discomfort at the medial side of the midfoot.

The patient was able to return to work at 10 months postsurgery. He reported mild discomfort at the medial side of the midfoot while wearing his shoes. This was related to an osseous fragment next to the N-C1 joint. Conventional weight-bearing X-rays at 10 months show anatomical alignment (Figure 6). Surgery has been planned to remove hardware, as well as this bony fragment.

## 3. Discussion

Among traumatic injuries to the midfoot, true dislocations are uncommon [3]. Moreover, the majority of injuries affect mainly the tarsometatarsal (Lisfranc) joint, followed by the midtarsal (Chopart) joint, and combined lesions are rare [5]. We reported the case of an unusual Chopart dislocation, since the calcaneocuboid joint was affected, but not the talonavicular joint. Instead, the deforming force went through the naviculocuneiform joint. To our knowledge, only 5 cases with this pattern of dislocation have been described in the literature [13–16]. All were due to crush injury or high-energy trauma. Cheng et al. [13] reported two cases treated with open reduction and internal fixation, whereas the other authors described closed reduction with percutaneous pinning [14, 15] or closed reduction alone [16].

In our case, we decided to perform open reduction after an incomplete reduction was visualized on the CT scan following closed manipulation and percutaneous pinning. Moreover, Richter et al. [17] showed that anatomical reduction was essential for good outcomes in Chopart injuries and that open reduction resulted in higher scores/outcomes than closed reduction. This was also described by Myerson et al. [18], knowing that reconstruction of the foot and maintaining articular congruency are critical. Regarding the surgical strategy, the first essential step is to obtain stability of the medial column [4, 19]; therefore, we addressed the naviculocuneiform joint before the calcaneocuboid joint. This was performed using a dorsomedial approach, from the talar neck to the base of the second metatarsal, allowing access to the first and second cuneiforms, through intervals on both sides of the extensor hallucis longus tendon [4]. After stabilization of the medial column, the calcaneocuboid joint was not anatomically reduced. Knowing that this joint is considered an essential joint because of the motion needed to accommodate to uneven ground [4, 6], we chose to address the joint with a second classic lateral approach [20] and to stabilize the anatomical reduction with Kirschner wires, in order to facilitate removal of hardware at 6 weeks postoperatively.

The choice between open reduction internal fixation (ORIF) and primary arthrodesis is controversial regarding traumatic injuries of the midfoot [21], and most studies are related to Lisfranc injuries [22–24], showing a trend towards better outcomes following primary arthrodesis. Very few data are available in the literature concerning outcomes after arthrodesis for Chopart injuries. Rammelt and Schepers [25] described significantly worse results with primary fusion compared to ORIF. Grambart et al. [7] described two cases of naviculocuneiform dislocations, treated with primary arthrodesis; this decision was based on severe osteochondral injury to the navicular. In our case, we could have performed a primary arthrodesis of the naviculocuneiform joint, knowing that this is a nonessential joint with minimal motion [4]. However, given the patient's age and the quality of articular surfaces, we decided to avoid primary fusion. Of note, Roling et al. [26] demonstrated in their cadaveric study that the naviculocuneiform joint contributes to 50% of the range of motion of the first ray and that arthrodesis of this joint reduces the motion of 40%. Moreover, some studies found that ORIF provides fewer complications and lower costs [21], with no significant difference in clinical outcomes [27], compared to primary arthrodesis for midfoot injuries.

In conclusion, we presented a case of unusual dislocation of the midfoot, treated with open reduction with good short-term result. The follow-up was not long enough to state that ORIF is superior to primary arthrodesis for this patient. However, this case illustrated the critical importance of anatomical reduction of midfoot injuries to obtain favorable outcomes, as described in the literature.

## Data Availability

Complete data are available on request to the corresponding author.
